# 177. Distinctive Features of Ertapenem Mono-Resistant Carbapenem-Resistant Enterobacterales in the United States: A Cohort Study

**DOI:** 10.1093/ofid/ofab466.177

**Published:** 2021-12-04

**Authors:** Max W Adelman, Chris W Bower, Julian E Grass, Uzma Ansari, Isaac See, Joseph D Lutgring, Jesse T Jacob

**Affiliations:** 1 Emory University School of Medicine, Atlanta, Georgia; 2 Georgia Emerging Infections Program, Decatur, GA; 3 Division of Healthcare Quality Promotion, Centers for Disease Control and Prevention, Atlanta, GA; 4 Centers for Disease Control and Prevention, Atlanta, Georgia; 5 Emory University, Atlanta, GA

## Abstract

**Background:**

Carbapenem-resistant Enterobacterales (CRE) are highly antibiotic-resistant bacteria. Whether CRE resistant only to ertapenem among carbapenems (ertapenem mono-resistant) represent a unique CRE subset with regards to risk factors, carbapenemase genes, and outcomes is unknown.

**Methods:**

We analyzed laboratory- and population-based surveillance data from nine sites participating in CDC’s Emerging Infections Program (EIP). We defined an incident case as the first isolation of *Enterobacter cloacae *complex, *Escherichia coli*, *Klebsiella aerogenes*, *K. oxytoca*, *K. pneumoniae,* or *K. variicola* resistant to doripenem, ertapenem, imipenem, or meropenem (determined at clinical laboratory) from a normally sterile site or urine identified from a resident of the EIP catchment area in 2016-2017. We compared risk factors, carbapenemase genes (determined via polymerase chain reaction at CDC), and mortality of cases with ertapenem “mono-resistant” to “other” CRE (resistant to ≥ 1 carbapenem other than ertapenem). We additionally conducted survival analysis to determine the effect of ertapenem mono-resistant status and isolate source (sterile vs. urine) on survival.

**Results:**

Of 2009 cases, 1249 (62.2%) were ertapenem mono-resistant and 760 (37.8%) were other CRE (**Figure 1**). Ertapenem mono-resistant CRE cases were more frequently ≥ 80 years old (29.1% vs. 19.5%, p< 0.0001), female (67.9% vs 59.0%, p< 0.0001), and white (62.6% vs. 45.1%, p< 0.0001). Ertapenem mono-resistant isolates were more likely than other CRE to be *Enterobacter cloacae *complex (48.4% vs. 15.4%, p< 0.0001) but less likely to be isolated from a normally sterile site (7.1% vs. 11.7%, p< 0.01) or have a carbapenemase gene (2.4% vs. 47.4%, p< 0.0001) (**Figure 2**). Ertapenem mono-resistance was not associated with difference in 90-day mortality (unadjusted odds ratio [OR] 0.82, 95% confidence interval [CI] 0.63-1.06) in logistic models or survival analysis (**Figure 3**).

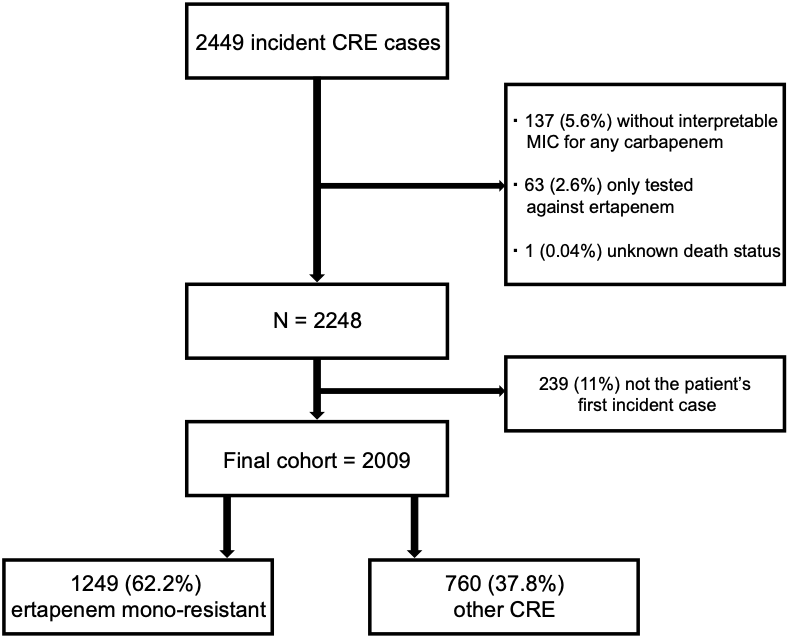

Figure 1. Flow diagram of carbapenem-resistant Enterobacterales cases included in analysis, 2017-2018. CRE, carbapenem-resistant Enterobacterales; MIC, minimum inhibitory concentration. Ertapenem mono-resistant CRE are only resistant to ertapenem (among carbapenems). Other CRE are resistant to ≥1 carbapenem other than ertapenem. We excluded isolates that (1) had no interpretable MICs for any carbapenem, (2) were only tested against ertapenem, (3) had unknown death status, or (4) were not associated with patient’s first incident case.

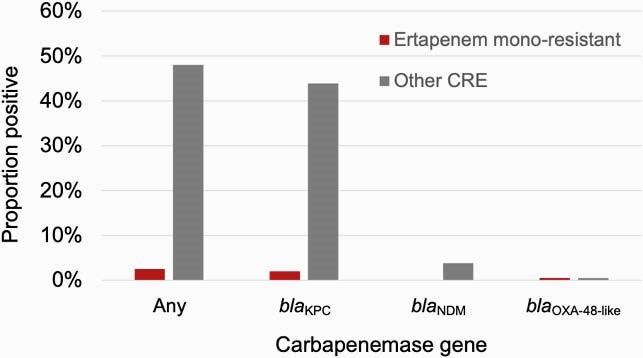

Figure 2. Proportion of ertapenem mono-resistant carbapenem-resistant Enterobacterales (CRE) vs. other CRE isolates with specific carbapenemase genes. KPC, Klebsiella pneumoniae carbapenemase; NDM, New Delhi metallo-ß-lactamase; OXA, oxacillinase. Ertapenem mono-resistant carbapenem-resistant Enterobacterales (CRE) are only resistant to ertapenem (among carbapenems). Other CRE are resistant to ≥1 carbapenem other than ertapenem. Testing via reverse transcriptase polymerase chain reaction.

Figure 3. Survival analysis comparing patients with carbapenem-resistant Enterobacterales (CRE) that are ertapenem mono-resistant to other CRE (i.e., resistant to ≥1 carbapenem other than ertapenem), either total (A) or stratified by isolate site (B).

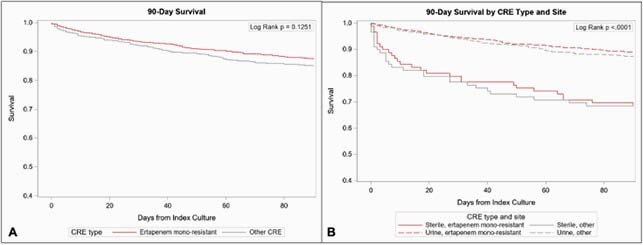

Ertapenem mono-resistant) isolates were not associated with decreased mortality, and sterile isolate source (i.e., non-urinary isolates) was associated with increased mortality regardless of ertapenem mono-resistance.

**Conclusion:**

Ertapenem mono-resistant CRE rarely have carbapenemase genes and have distinct clinical and microbiologic characteristics compared to other CRE. These findings may inform antibiotic choice particularly when testing for carbapenemases is not readily available.

**Disclosures:**

**All Authors**: No reported disclosures

